# Efficacy and Safety of Zhenwu Decoction in the Treatment of Diabetic Nephropathy: A Systematic Review and Meta-Analysis

**DOI:** 10.1155/2022/2133705

**Published:** 2022-11-01

**Authors:** Xialin Lv, Min Zhou, Xiu Liu, Qin Xiang, Rong Yu

**Affiliations:** ^1^Hunan University of Chinese Medicine, Changsha 410208, China; ^2^Hunan Provincial Key Laboratory of Translational Research in Traditional Chinese Medicine Prescriptions and Zheng, Changsha 410208, China

## Abstract

**Objective:**

To perform a systematic evaluation of the clinical efficacy and safety of Zhenwu decoction (ZWD) for the treatment of diabetic nephropathy (DN).

**Methods:**

PubMed, the China National Knowledge Infrastructure (CNKI), the China Science and Technology Journal Database (VIP), the Chinese Biomedical Literature Database (CBM), and the WanFang databases were searched, and a systematic review and meta-analysis of randomized controlled trials (RCTs) were subsequently conducted to compare the efficacy and safety of ZWD combined with conventional Western medicine (CWM) to conventional therapy alone in the treatment of DN. The Cochrane Handbook for Systematic Reviews of Interventions and GRADE criteria were utilized to assess the quality of the included literature, and RevMan 5.3 software was used for statistical analysis.

**Results:**

13 randomized controlled trials were included, involving 1347 patients with diabetic nephropathy assigned into two subgroups according to the disease duration. The results revealed that compared with conventional therapy alone, ZWD combined with CWM treatment significantly improved the total effective rate (OR = 3.88, 95% CI = (2.87, 5.26), *P* < 0.00001). Furthermore, ZWD combination therapy also decreased fasting blood glucose (MD = −0.72, 95% CI = (−0.97, −0.48), *P* < 0.00001), BUN (MD = −1.92, 95% CI = (−3.19, −0.64), *P* = 0.003), 24-hour urine protein (MD = −0.48, 95% CI = (−0.57, −0.39), *P* < 0.00001), and serum creatinine levels (MD = −51.17, 95% CI = (−66.95, −35.39), *P* < 0.00001). However,there was no statistical significance in the effect of combination therapy on creatinine clearance (MD = −0.64, 95% CI = [−8.21,6.92], P = 0.87). However, there was no statistical significance in the effect of combination therapy oncreatinine clearance (MD =−0.64, 95% CI=[−8.21,6.92], *P*=0.87).

**Conclusion:**

ZWD combined with CWM outperformed conventional Western medicine in DN treatment. However, further investigations via multicenter RCTs with rigorous designs and higher quality are still warranted.

## 1. Introduction

According to the World Health Organization (WHO), the incidence of diabetes in China reached 11.2% in 2017 (compared to 0.67% in 1980), with approximately 114 million individuals suffering from diabetes and accounting for 24% of the total number of patients [1]; this was higher than the global incidence of 8.4% [2]. The International Diabetes Federation (IDF) estimates that there could be 578 million people with diabetes worldwide (10.2%) by 2030 [3] and 783.2 million (12.2%) by 2045 [4]. Diabetic nephropathy (DN), one of the major complications of diabetes, is the leading cause of end-stage renal disease (ESRD). Based on the global prevalence of diabetes, the incidence of DN is increasing. Surveys indicate that approximately 40% of patients with type 2 diabetes mellitus (T2DM) are likely to develop DN [5].

The predominant pathological characteristics of DN consist of glomerular sclerosis, tubulointerstitial fibrosis, and renal angiopathy. Its pathogenic factors and pathogenesis are complex and are principally related to glycolipid metabolism disorders, insulin resistance, hemodynamic fluctuations, oxidative stress, inflammation, endoplasmic reticulum stress, autophagy, exosomes, and intestinal flora; however, the specific mechanism remains to be further clarified [6–10]. Clinically, proteinuria, renal function, and diabetes history are considered the main diagnostic indicators of DN. Presently, there is no specific drug for treating DN, and management mainly includes controlling blood sugar and blood pressure, reducing proteinuria, and supporting symptomatic treatment [11], which fail to prevent disease progression. Therefore, the integration of traditional Chinese and Western medicine in the treatment of DN has garnered increasing attention.

Extensive investigation of the various pathological mechanisms of DN has revealed that the clinical efficacy of single-target therapy is suboptimal. Due to the limitations of applying such treatment, a higher proportion of studies are dedicated to investigating combination therapy. Unlike conventional Western medicine (CWM), traditional Chinese medicine (TCM) prescriptions combine a variety of medicinal materials in specific proportions based on TCM's theoretical underpinnings. There are hundreds of potential chemical components exist in the formula. Some bioactive chemicals that can simultaneously act on several targets for treatment have been identified. When combined, these active ingredients interact synergistically or antagonistically to modulate each other and yield a favorable therapeutic effect.

Records of TCM being used to treat DN in ancient China can be traced back to 2000 years ago. Based on its clinical manifestations, DN can be categorized as “xiaoke”. With the concurrent occurrence of hypertension, proteinuria, edema, and other diseases, further classifications such as “shenxiao,” “edema,” and “guange” can be associated with DN. Modern Chinese medicine also refers to DN as “xiaoke nephropathy”. Zhenwu decoction (ZWD) is derived from the “Treatise on Febrile Diseases” (Shanghan Zabing Lun in China) by Zhang Zhongjing, which dates back to the Eastern Han Dynasty. It comprises five herbs: *Aconiti Lateralis Radix Praeparata*, *Poria*, *Atractylodis Macrocephalae Rhizoma*, *Paeoniae Radix Alba*, and *Zingiberis Rhizoma Recens* (Table 1). These herbs have the combined effect of invigorating the spleen, tonifying the kidney, warming the yang, and alleviating water retention. ZWD is utilized to treat yang deficiency arising from yang deficiency in the spleen and kidney. Subsequent generations of physicians have conducted extensive research in this field and have been attempting to treat DN on the basis of this prescription.

Due to its definite therapeutic effect and scarce side effects, ZWD has been widely adopted in clinical settings. Despite an increasing number of clinical reports on combining ZWD and conventional western medicine for DN treatment, there is limited evidence of its effectiveness and safety. Therefore, a comprehensive and systematic evaluation of this combination drug is crucial. This study aims to provide theoretical and evidence-based medical support for the treatment of DN by conducting a meta-analysis to evaluate ZWD's effectiveness and safety.

## 2. Materials and Methods

The review protocol was conducted under the guidance of PRISM and registered on International Platform of Registered Systematic Review and Meta-analysis Protocols (INPLASY) with the registration number of INPLASY202290071.

### 2.1. Data Sources and Search Strategy

To identify the clinical studies on ZWD combined with CWM for the treatment of DN, we searched five databases from their inception to February 2022: PubMed, the China National Knowledge Infrastructure (CNKI), the China Science and Technology Journal Database (VIP), the Chinese Biomedical Literature Database (CBM), and the WanFang databases. The following keywords were used: “Zhen Wu Decoction,” “Zhen-Wu-Decoction,” “Traditional Chinese medicine”, “Chinese herb medicine,” “Diabetic Nephropathy,” “Diabetes Mellitus,” “type 2 Diabetes Mellitus,” “T2DM”, “Diabetic Kidney Disease,” “Kidney Diseases,” or “randomized controlled trial,” “Randomized,” “clinical research,” and “placebo”. The data were independently studied and collated by the two authors, and manual searches were conducted to track the necessary references and further improve the relevant information. Subsequent to this process, the target research articles were finally confirmed.

### 2.2. Eligibility and Exclusion Criteria

#### 2.2.1. Eligibility Criteria

The eligibility criteria are as follows;

(1) *Study type*. Randomized controlled trials (RCTs) published in Chinese and English on ZWD for diabetic nephropathy.

(2) *Type of participants*. Adult patients who met the diagnostic criteria of DN.

(3) *Intervention measures*. The control group was treated with CWM, including diabetes medication, hypoglycemic drugs, and hypotensive drugs. The experimental group was administered either add-on ZWD in conjunction with the control group treatment or ZWD alone.

*(4) Outcome indicators*. The clinical efficacy (total effective rate), fasting blood glucose (FBG), blood urea nitrogen (BUN), 24-hour urine protein, creatinine clearance (Ccr), and serum creatinine (Scr).

#### 2.2.2. Exclusion Criteria

The exclusion criteria are as follows: (1) non-RCT; (2) no control group; (3) the experimental group adopt with other therapeutic methods, except for ZWD + CWM treatment or ZWD alone; (4) the control group was not treated with CWM; (5) nondiabetic nephropathy; (6) they did not meet the DN diagnostic criteria or did not clearly describe the diagnostic criteria; (7) the subjects suffered from severe primary diseases; (8) duplicated detection or published literature; (9) no target outcomes; (10) missing data and unable to contact the investigator.

### 2.3. Data Collection

The two system reviewers independently conducted extensive screening of the preliminary research articles potentially meeting the inclusion criteria by examining titles and abstracts and eliminated nonconforming literature. Afterward, the two reviewers cross-checked the included documents and examined the full text to extract target data for classification and integration. If differing opinions arose, a third researcher (Rong Yu) was consulted. The extracted data included the authors, publication year, baseline data (i.e., sample size, age, and duration), intervention measures, outcome indicators, and adverse events.

### 2.4. Quality Assessment

The methodological quality of the included RCTs was evaluated based on the assessment criteria outlined in the Cochrane Systematic Review Manual. The quality criteria included the following: accuracy of the random allocation method; adequacy of allocation concealment; use of blinding methods; patients who were lost to follow-up or withdrew from the study; integrity of the outcome data; and other biases. GRADE prosoftware was utilized to assess the strength of the evidence to enhance the results' validity.

### 2.5. Statistical Analysis

The meta-analysis was performed using Review Manager 5.3.3 and Stata 12.0 software. We used the odds ratio (OR) to assess the binary variables. For continuous variables, the mean difference (MD, when results were in similar units of measure) or standardized mean difference (SMD, when results were in different units of measure) were employed to represent the difference between the groups. The results were represented with a 95% confidence interval (CI). The heterogeneity was evaluated using the chi-square test; if *P* > 0.1 or I^2^ <50%, it was assumed that the heterogeneity was not evident and the fixed-effects model was selected; otherwise, the random effects model was validated. In addition, a sensitivity analysis was performed for each outcome to assess stability. We also completed the Egger test to detect potential publication bias.

## 3. Results

### 3.1. Literature Search Results and Study Characteristics

**Figure 1:**
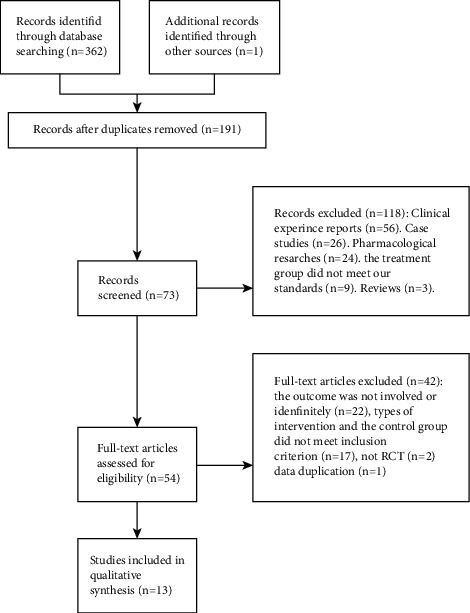
Flow diagram of the literature selection process.

As presented in Figure 1, 362 research articles were collected via the retrieval strategy, 191 duplicate studies were removed, and after the multilayer screening, 13 articles were eventually included [12–24]. A total of 1347 DN patients were identified, with the control group (treated with CWM) and experimental group (ZWD alone or combined with CWM treatment) comprising 671 and 676 patients, respectively. The specific details of the included studies are displayed in Table 2.

### 3.2. Risk-of-Bias Assessment

**Figure 2:**
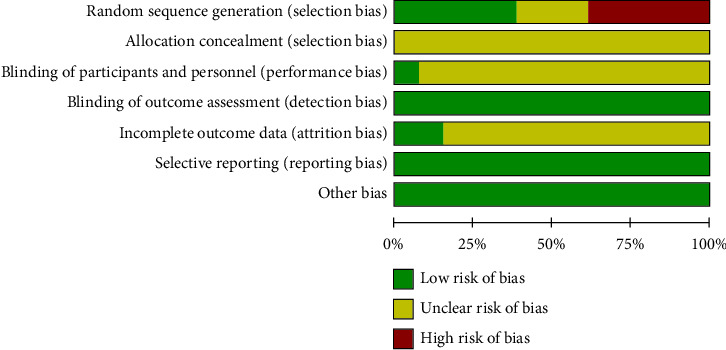
Risk of bias graph.

**Figure 3:**
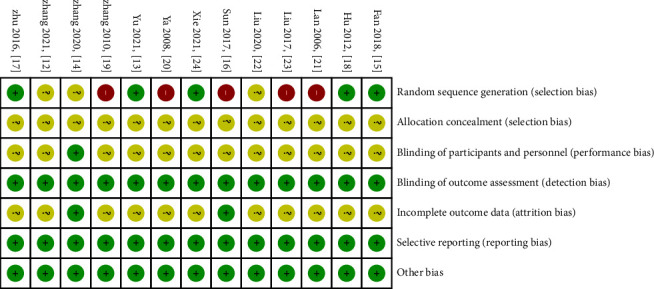
Risk of bias summary.

In the included literature, 11 articles mentioned a random method; of those, six reported specific randomization methods (including a random number table method, randomization by visit order) [13, 15, 17, 18, 22, 24], while the remaining five did not report specific randomization methods [16, 19–21, 23]. Two studies did not mention randomization [12, 14]. None of the articles reported allocation concealment, and one article reported double-blind method implementation [14]. Two articles reported that no adverse reactions occurred [14, 16]. The detailed methodological quality evaluation is presented in Figures 2 and 3.

### 3.3. Meta-Analysis Result

#### 3.3.1. Total Effective Rate

Preliminary statistics indicated that total efficacy was disclosed in all included articles [12–24]. Heterogeneity was not evident (*P* = 0.98, I^2^ = 0%) when a fixed-effects model was applied. The results were indicative of a statistically significant higher total effective rate in the experimental group compared to the control group (OR = 3.88, 95% CI = [2.87, 5.26], *P* < 0.00001). Hence, for the treatment of diabetic nephropathy, the combination of ZWD and CWM outperformed CWM alone.

**Figure 4:**
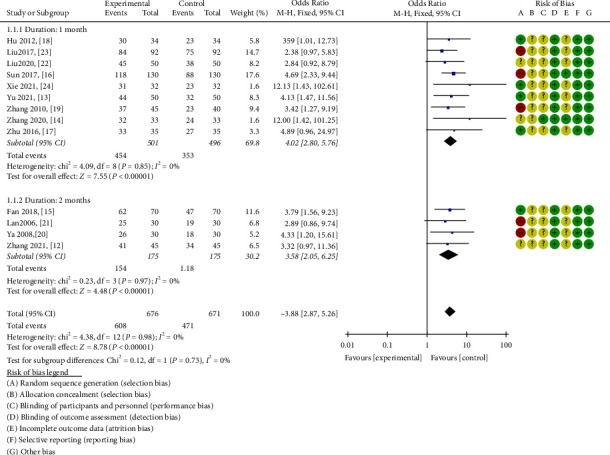
Forest plots of ZWD on total effective rate.

An additional subgroup analysis demonstrated the superior efficacy of ZWD compared to the control group for treatment lasting one month [13, 14, 16–19, 22–24] (chi-square = 4.09, I^2^ = 0%, OR = 4.02, 95% CI = [2.80, 5.76], *P* < 0.00001) and two months [12, 15, 20, 21] (chi-square = 0.23, I^2^ = 0%, OR = 3.58, 95% CI = [2.05, 6.25], *P* < 0.00001) (Figure 4).

#### 3.3.2. FBG

12 studies were included in the fasting blood glucose analysis. The random effects model was selected based on the heterogeneity test results (*P* < 0.00001, I^2^ = 76%). Compared with the control group, ZWD significantly reduced fasting blood glucose and improved glucose metabolism in DN patients (MD = −0.72, 95% CI = (−0.97, −0.48), *P* < 0.00001).

**Figure 5:**
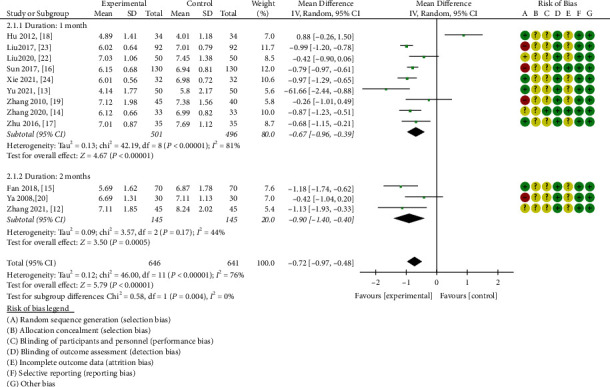
Forest plots of ZWD on fasting blood glucose.

The subgroup analysis revealed that the ZWD group's hypoglycemic effect surpassed that of the control group, irrespective of whether the duration was one month [13, 14, 16–19, 22–24] (chi-square = 42.19, I^2^ = 81%, MD = −0.67, 95%CI = [−0.96,−0.39], *P* < 0.00001) or two months [12, 15, 20] (chi-square = 3.57, I^2^ = 44%, MD = −0.90, 95%CI = [−1.40,−0.40], *P* = 0.0005) (Figure 5).

#### 3.3.3. BUN

**Figure 6:**
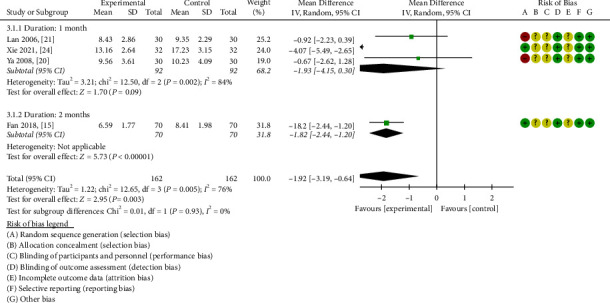
Forest plots of ZWD on blood urea nitrogen.

As depicted in Figure 6, four studies encompassing 324 patients were included, with patients divided 1 : 1 between the control and experimental groups [15, 20, 21, 24]. A random effects model was implemented for statistical analysis based on the heterogeneity test (*P* = 0.005, I^2^ = 76%). The meta-analysis results indicated that ZWD possessed a higher propensity to reduce BUN compared to CWM alone (MD = −1.92, 95%CI = [−3.19,−0.64], *P* = 0.003) (Figure 6).

A subsequent subgroup analysis revealed evident heterogeneity (chi-square = 12.50, I^2^ = 84%) during the one-month treatment course. Application of the random effects model demonstrated that there was no statistical significance between the two study groups (MD = −1.93, 95%CI = [−4.15, 0.30], *P* = 0.09) [20, 21, 24]. Despite undetectable heterogeneity during the two-month treatment course, statistical analysis established that ZWD combined with CWM was superior to the control group (MD = −1.82, 95% CI[−2.44,−1.20], *P* < 0.00001) [15].

#### 3.3.4. 24-Hour Urine Protein

Eight articles [13–16, 20, 21, 23, 24] focused on 24-hour urinary protein level fluctuations. The heterogeneity was apparent (chi-square = 16.74, *P* = 0.02, I^2^ = 58%); therefore, the random effects model was selected. In patients with DN, ZWD significantly reduced 24-hour urinary protein levels compared with CWM (MD = −0.48, 95% CI = [−0.57,−0.39], *P* < 0.00001).

**Figure 7:**
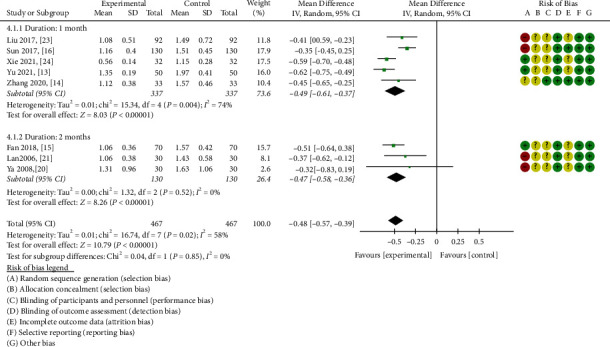
Forest plots of ZWD on 24 h urine protein.

A subgroup analysis indicated significant heterogeneity (chi-square = 15.34, *P* = 0.004, I^2^ = 74%) during the one-month treatment course [13, 14, 16, 23, 24]. On the basis of the random effects model, ZWD treatment of DN was determined to induce a statistically significant effect compared with CWM treatment (MD = −0.49, 95% CI = [−0.61,−0.37], *P* < 0.00001). For the two-month treatment course [15, 20, 21], heterogeneity was not readily apparent (chi-square = 1.32, *P* = 0.52, I^2^ = 0%), and the statistical analysis indicated that ZWD combined with CWM was superior to the control group in terms of improving 24-hour urine protein levels (MD = −0.47, 95%CI = [−0.58,−0.36], *P* < 0.00001) (Figure 7).

#### 3.3.5. Creatinine Clearance

**Figure 8:**
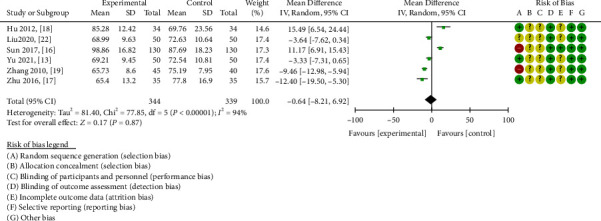
Forest plots of ZWD on creatinine clearance.

A total of six research articles thoroughly investigated alterations in serum creatinine clearance [13, 16–19, 22]. The overall heterogeneity was manifested (chi-square = 77.85, *P* < 0.00001, I^2^ = 94%); hence, the random effects model was selected. In patients with DN, the combination of ZWD and CWM was not statistically significant in enhancing the serum creatinine clearance compared to CWM alone (MD = -0.64, 95%CI = [-8.21,6.92], *P* =0.87 ). (Figure 8).

#### 3.3.6. Scr

Eight eligible studies were included to analyze the Scr outcome [14–16, 18, 20, 21, 23, 24], with a total of 902 patients distributed evenly across the two study groups. Based on the observed heterogeneity (*P* < 0.00001, I^2^ = 81%), the random effects model was selected for analysis. ZWD significantly decreased serum creatinine levels in DN patients compared with the control group (MD = −51.17, 95%CI = [−66.95,−35.39], *P* < 0.00001).

**Figure 9:**
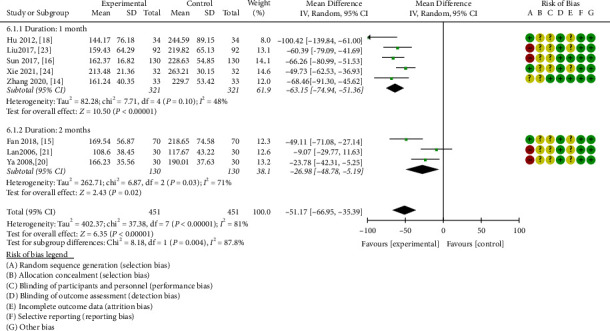
Forest plots of ZWD on serum creatinine.

A detailed subgroup analysis illustrated that compared with the control group, ZWD could significantly reduce serum creatinine levels in the one-month (MD = −63.15, 95%CI = [−74.94,−51.36], *P* < 0.00001) [14, 16, 18, 23, 24] and two-month treatment courses (MD = −26.98, 95%CI = [−48.78,-5.19],*P* = 0.02) [15, 20, 21] (Figure 9).

#### 3.3.7. Sensitivity Analysis and Publication Bias

**Figure 10:**
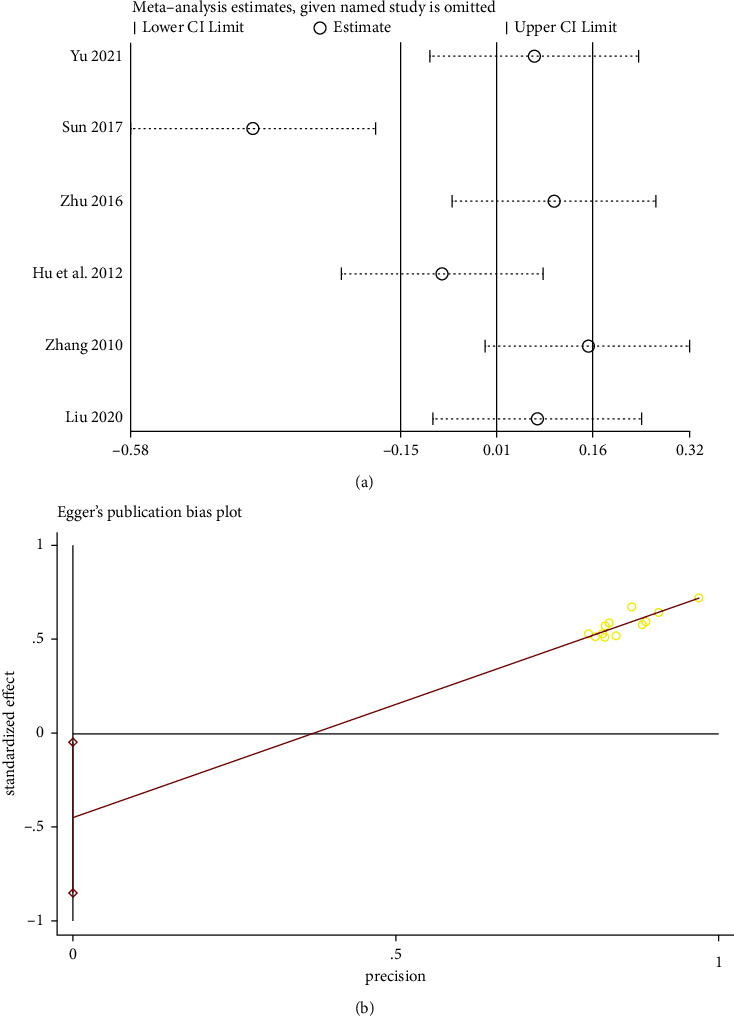
(a) Sensitivity analysis for the creatinine clearance. (b) Egger test of the total effective rate.

An item-by-item elimination method was applied to investigate the included literature's data for a sensitivity analysis of the total effective rate, FBG, BUN, 24 h urine protein, creatinine clearance, and Scr. There were no significant changes in the stability of each study and the aggregated results of each effect size, except creatinine clearance, indicating the validity of the data analysis results. Furthermore, Egger's test was performed for each outcome to assess the potential publication bias. *P* < 0.05 was indicative of publication bias. The analysis revealed the absence of publication bias for indicators other than the total effective rate (*P* = 0.032 < 0.05). The creatinine clearance and the effective rate are depicted in Figure 10, and the entire summary is provided in Table 3. An extensive literature search revealed that all studies were conducted in China and that all reported results were favorable. The publication bias may be related to the region, race, and unpublished negative results.

#### 3.3.8. Evidence Quality Rating of Outcome Indicators

Evidence quality was evaluated using the GRADE prosoftware; the majority of outcome indicators possessed moderate reliability, while one outcome indicator was graded as low-quality evidence (Table 4).

## 4. Discussion

In this study, ZWD significantly diminished fasting blood glucose, BUN, 24-hour urine protein, and serum creatinine levels; improved total effective rate, except for creatinine clearance(P＞0.05), with no noteworthy adverse effects. This demonstrates that ZWD is a safe and effective renoprotective therapeutic option. Moreover, it can be seen that in the future clinical treatment of DN, ZWD will be further popularized. Our findings also augment confidence for an in-depth study of the mechanism of ZWD.

TCM is favored by numerous T2DM patients [25] and DN patients [26] by virtue of attributes such as a multi-target approach, low toxicity, and few side effects. Owing to its spleen-strengthening and yang-nourishing abilities, Zhenwu decoction has been a mainstay of Chinese clinical practice for thousands of years. According to modern pharmacological studies on its components, (1) ACPP-1, a polysaccharide derived from *Aconitum coreanum (fuzi)*, markedly inhibits *α*-glycosidase and reduces the serum glucose level [27]. (2) Polysaccharides extracted from *Atractylodes macrocephala* reduce fasting blood glucose in type 2 diabetic mice, improve glucose tolerance, enhance insulin sensitivity [28], and are endowed with diuretic and anti-inflammatory properties [29, 30]. (3) Pachymic acid (PA), an extractive derived from *Poria*, decreases serum creatinine and blood urea nitrogen and alleviates renal pathological damage in mice with acute kidney injury [31]. Poria polysaccharide decreases 24 h urine protein and serum creatinine, averts kidney damage in type 2 diabetic rats, and impedes the development of diabetic nephropathy to a certain extent [32]. It also possesses antioxidant, anti-inflammatory, and renoprotective attributes [33]. (4) Curcumin from *Zingiberis Rhizoma Recens* diminishes blood glucose, Scr, blood urea nitrogen, and urine albumen levels in DN rats. It regulates autophagy, attenuates epithelial-to-mesenchymal transition via the PI3k/Akt/mTOR pathway [34], and ameliorates DN in rats by alleviating renal inflammation and oxidative stress [35]. In one clinical study, curcumin significantly lessened proteinuria in patients with DN [36]. (5) Paeoniflorin regulates macrophages by inhibiting the iNOS expression and inflammatory factor production, thereby mitigating clinical symptoms and diminishing the occurrence of DN in mice [37]. Previous studies demonstrated that paeoniflorin alleviates damage to glomerular mesangial cells via the RAGE/mTOR autophagy pathway [38]. Its active ingredients reduce proinflammatory factor release through the endoplasmic reticulum stress pathway [39].

Contemporary studies have also demonstrated that ZWD can improve proteinuria and renal damage in rats with streptozotocin-induced diabetic nephropathy [40], alleviate cisplatin-induced acute kidney injury [41], protect against IgA nephropathy by regulating exosomes to inhibit the NF-kB/NLRP3 pathway [42], and mitigate podocyte injury in rats with IgA nephropathy through the PPAR*γ*/NF-*κ*B pathway [43].

The present study had several limitations. Firstly, the allocation concealment method was not defined in all of the included articles, and the accuracy of the collected clinical research data has yet to be verified. Secondly, TCM treatment of diseases is “syndrome-”based, and syndrome differentiation is the fundamental guiding principle of TCM intervention. However, in clinical research, researchers usually apply a specific drug to the treatment of DN, resulting in feeble or nonexistent syndrome differentiation and treatment. Thirdly, the efficacy of DN intervention is principally reflected in the longer time period following the intervention, making the evaluation of long-term efficacy particularly vital. However, follow-up observations of the long-term efficacy of patients in clinical studies are generally lacking and limited to the short-term time period following drug intervention. Finally, the dearth of multicenter and large-sample size prospective randomized controlled trials in clinical research diminishes the reliability and credibility of the experimental data. Therefore, more multicenter prospective studies with a large-sample size should be performed in subsequent clinical research.

## 5. Conclusion

In conclusion, compared with conventional Western medicine therapy, combination therapy can increase the total effective rate of DN patients and reduce fasting blood glucose, BUN, 24-hour urinary protein, and serum creatinine levels. Our results indicate that ZWD can impede the progression of DN by ameliorating glucose metabolism and renal function. This review provides a theoretical basis for the clinical application of ZWD combined with CWM in the treatment of DN. However, more high-quality multicenter RCTs would be required to validate the conclusions further and guide clinical practice.

## Figures and Tables

**Table 1:** The prescription of Zhenwu decoction.

Prescription/herbs	Scientific names	Families
Poria	*Poria cocos (Schw.) Wolf*	Polyporaceae
Paeoniae radix alba	*Paeonia lactiflora Pall.*	Ranunculaceae
Zingiberis rhizoma recens	*Zingiber officinale Rosc.*	Zingiberaceae
Aconiti lateralis radix praeparata	*Aconitum carmichaelii Debx*.	Ranunculaceae
Atractylodis macrocephalae rhizoma	Atractylodes macrocephala koidz.	Asteraceae

**Table 2:** Details of the included studies.

No.	Authors, publication year	Sample sizes (M/F)	Mean age (years)	Duration (months)	Intervention		Adverse events	Outcomes
					Experimental group	Control group		
1	Zhang et al. 2021 [12]	E:23/22 C:24/21	E:67.9 ± 10.1 C:67.6 ± 10.3	2	ZWD + *C*	Hypoglycemic drugs	Not mentioned	①②

2	Yu 2021 [13]	E:31/19 C:33/17	E:57.94 ± 8.87 C:58.21 ± 8.66	1	ZWD + *C*	INS	Not mentioned	①②④⑤

3	Zhang 2020 [14]	E:18/15 C:17/16	E:54.8 ± 4.5 C:54.6 ± 4.2	1	ZWD + *C*	Hypoglycemic drugs/INS	Not found	①②④⑥

4	Fan 2018 [15]	E:40/30 C:38/32	E:63.57 ± 4.25 C:63.26 ± 4.33	2	ZWD + *C*	Hypoglycemic drugs/INS	Not mentioned	①②③④⑥

5	Sun 2017 [16]	E:68/62 C:64/66	E:46.82 ± 7.65 C:45.69 ± 7.26	1	ZWD + *C*	Hypoglycemic drugs/INS	Not found	①②④⑤⑥

6	Zhu 2016 [17]	E:19/16 C:18/17	E:58.4 ± 5.5 C:58.5 ± 5.6	1	ZWD + *C*	Hypoglycemic drugs/INS	Not mentioned	①②⑤

7	Hu et al. 2012 [18]	E:24/10 C:22/12	E:46.5 ± 10.8 C:47.8 ± 12.3	1	ZWD + *C*	INS	Not mentioned	①②⑤⑥

8	Zhang 2010 [19]	E:25/20 C:23/17	Not mentioned	1	ZWD + *C*	INS	Not mentioned	①②⑤

9	Ya and wang 2008 [20]	E:30 C:30 (39/21)	36–66	2	ZWD + *C*	Hypoglycemic drugs + hypotensor (captopril)	Not mentioned	①②③④⑥

10	Lan et al. 2006 [21]	E:13/17 C:14/16	18–65	2	ZWD + *C*	Hypoglycemic drugs + hypotensor (captopril)	Not mentioned	①③④⑥

11	Liu and Hu 2020 [22]	E:36/14 C:35/15	E:57.35 ± 8.77 C: 56.37 ± 9.12	1	ZWD + *C*	INS	Not mentioned	①②⑤

12	Liu 2017 [23]	E:49/43 C:40/52	E:49.5 ± 17.5 C:53.5 ± 18.5	1	ZWD + *C*	CWM	Not mentioned	①②④⑥

13	Xie 2021 [24]	E:19/13	E:51.32 ± 2.15 C:51.26 ± 2.28	1	ZWD + *C*	Hypotensor (losartan)	Not mentioned	①②③④⑥

① Total effective rate ② fasting blood glucose (FBG) ③ blood urea nitrogen (BUN) ④ 24h urine protein ⑤ creatinine clearance (Ccr) ⑥ serum creatinine (Scr) ZWD: ZhenWu decoction C: control group INS: insulin CWM: conventional Western medicine.

**Table 3:** Summary of sensitivity analysis and publication bias.

	OR/MD fluctuations	95%CI fluctuations	Publication bias (*P* value)
The effective rate	0.89	(0.83, 0.96)	0.032
FBG	−0.74	(−0.86, −0.63)	0.226
BUN	−0.75	(−0.98, −0.53)	0.744
24 h urine protein	−1.00	(−1.13, −0.86)	0.202
Ccr	0.00	(−0.14, 0.16)	0.168
Scr	−0.98	(−1.11, −0.84)	0.740

**Table 4:** Evidence quality for ZWD combined with CWM for the treatment of DN.

ZWD plus CWM for DN						
Patient or population: (patients with DN)						
Setting: all eligible patients with intervention therapy						
Intervention: (ZWD + CWM,CWM)						

Outcomes	Illustrative comparative risks*∗* (95% CI)		Relative effects (95% CI)	No. of participants (studies)	Quality of the evidence (GRADE)	Comments
	Assumed risk	Corresponding risk				
	Control	ZWD + CWM				

The effective rate	Study population		OR 3.88 (2.87 to 5.26)	1347 (13 studies)	⊕⊕⊕Ο moderate^ac^	
	199 per 1000	202 per 1000 (169 to 223)				
	Moderate					
	187per 1000	213 per 1000 (180 to 240)				

FBG	See comment	See comment	The mean was MD −0.72 lower (−0.97 to −0.48 lower)	1287 (12 RCTS)	⊕⊕⊕Ο moderate^ab^	

BUN	See comment	See comment	The mean was MD −1.92 lower (−3.19 to −0.64 lower)	324 (4RCTS)	⊕⊕⊕Ο moderate^ab^	

24 h urine protein	See comment	See comment	The mean was MD −0.48 lower (−0.57 to −0.39 lower)	934 (8 RCTS)	⊕⊕⊕Ο moderate^ab^	

Ccr	See comment	See comment	The mean was MD −0.64 lower (−8.21 to 6.92)	683 (6 RCTS)	⊕⊕ΟΟ low^abc^	

Scr	See comment	See comment	The mean was MD −51.17 lower (−66.95 to −35.39 lower)	902 (8 RCTS)	⊕⊕⊕Ο moderate^ab^	

*∗*The basis for the assumed risk (e.g., the median control group risk across studies) is provided in footnotes. The corresponding risk (and its 95% confidence interval) is based on the assumed risk in the comparison group and the relative effect of the intervention (and its 95% CI). CI: confidence interval; OR: odds ratio; GRADE: working group grades of evidence. High quality: further research is very unlikely to change our confidence in the estimate of the effect. Moderate quality: further research is likely to have an important impact on our confidence in the estimate of the effect and may change the estimate.Low quality: further research is very likely to have an important impact on our confidence in the estimate of the effect and is likely to change the estimate. Very low quality: we are very uncertain about the estimate. Explanations: (a) no blinding. (b) High heterogeneity. (c) *P* < 0.05 in Egger's test.

## Data Availability

The data used to support the findings of this study are available from the corresponding author upon request.
